# A Critical Review on Modification Methods of Cement Composites with Nanocellulose and Reaction Conditions during Nanocellulose Production

**DOI:** 10.3390/ma15217706

**Published:** 2022-11-02

**Authors:** Małgorzata Szafraniec, Ewelina Grabias-Blicharz, Danuta Barnat-Hunek, Eric N. Landis

**Affiliations:** 1 Faculty of Civil Engineering and Architecture, Lublin University of Technology, Nadbystrzycka 40, 20-618 Lublin, Poland; 2 Department of Civil and Environmental Engineering, University of Maine, Orono, ME 04469, USA

**Keywords:** cement composites, nanocellulose, nanomaterials, spherical nanocellulose, square/rectangular nanocellulose

## Abstract

Nanocellulose (NC) is a natural polymer that has driven significant progress in recent years in the study of the mechanical properties of composites, including cement composites. Impressive mechanical properties, ability to compact the cement matrix, low density, biodegradability, and hydrophilicity of the surface of nanocellulose particles (which improves cement hydration) are some of the many benefits of using NCs in composite materials. The authors briefly presented a description of the types of NCs (including the latest, little-known shapes), showing the latest developments in their manufacture and modification. Moreover, NC challenges and opportunities are discussed to reveal its hidden potential, as well as the use of spherical and square/rectangular nanocellulose to modify cement composites. Intending to emphasize the beneficial use of NC in cementitious composites, this article discusses NC as an eco-friendly, low-cost, and efficient material, particularly for recycling readily available cellulosic waste. In view of the constantly growing interest in using renewable and waste materials in a wide range of applications, the authors hope to provide progress in using nanocellulose (NC) as a modifier for cement composites. Furthermore, this review highlights a gap in research regarding the preparation of new types of NCs, their application, and their impact on the properties of cementitious composites. Finally, the authors summarize and critically evaluate the type, dosage, and application method of NC, as well as the effects of these variables on the final properties of NC-derived cement composites. Nevertheless, this review article stresses up-to-date challenges for NC-based materials as well as future remarks in light of dwindling natural resources (including building materials), and the principles of a circular economy.

## 1. Introduction

For decades, the practice has sought to improve the properties of cement using fibers of different origins and sizes. According to most researchers, cellulose fibers are non-toxic, generally available, renewable, and economical compared to other fibers such as polypropylene, but also show adequate bonding ability with cement matrices, thus improving their ductility, toughness, impact resistance, and flexibility [1,2,3,4,5]. However, cellulose also has disadvantages such as poor dispersion, long-term durability, and fiber mineralization. The use of nanostructured cellulosic materials—nanocellulose can contribute to the elimination of the disadvantages of cellulose fibers.

Cellulose is the most common biological polymer on earth, and due to its properties, it finds its application in engineering materials. Nanocellulose has gained a great deal of recognition among researchers thanks to its unique properties that combine the properties of cellulose and the unique characteristics of nanomaterials. In addition, it can be made from recycled materials such as cotton clothes or waste paper [6,7,8,9,10,11,12,13,14,15], which fits perfectly with the idea of sustainable construction and waste management. Because it is a biomass material, it is biodegradable, renewable, and environmentally friendly.

NC has several advantages over cellulose, including low weight, high strength, excellent stiffness, high surface area, and strong interaction with polymeric and inorganic compounds. The biodegradability, durability, and renewability of nanocellulose have attracted great interest in many technical and scientific fields. NC, as a material based on natural resources, has hundreds of potential applications in many industrial sectors. Nanocellulose can be produced by a top-down process, which includes the production of cellulose nanocrystals (CNCs) and cellulose nanofibers (CNFs), and a bottom-up process, which includes the synthesis of bacterial cellulose (BC) [16,17]. The top-down process involves the hydrolysis and isolation of CNCs or the mechanical reduction of fiber sizes to the nanoscale. In contrast, BC is produced by fermenting low-molecular-weight sugar using cellulose-producing bacteria. The massive variety of cellulose sources [18,19,20,21,22] and different ways of producing NC and its modification contribute to the formation of nanocellulose with different mechanical and physical properties and different morphology.

Cement composites have a complex structure that ranges from macro to nanoscale. Incorporating NC nanoparticles makes it possible to produce cement composites more resistant to frost and moisture, among other things, or composites with unique properties. The use of NC can reduce the need for synthetic fibers and help to improve the properties of composite materials.

The purpose of this review is to present the latest knowledge regarding reaction conditions during nanocellulose production, presentation of new types of nanocellulose that have not yet been applied to cement and concrete (including spherical nanocellulose), ways of modifying nanocellulose with other materials (nano-silica) and to present the latest ways to use nanocellulose in cement composites.

## 2. Cement and Cement Composites

Cement is a hydraulic binder with binding properties. It is obtained from raw mineral materials, specifically natural minerals such as marl, clay, or limestone. When mixed with water, it gives a slurry that hardens by reaction and hydration processes and retains its strength characteristics, even under water. The main component of cement is clinker, of which there are several types: Portland clinker used in manufacturing Portland cement, Aluminum clinker obtained from bauxite, calcium or bauxite, and limestone, Barium clinker obtained from raw materials containing calcium carbonates, barium, and aluminosilicates. Portland clinker is a basic intermediate product used in the cement industry. It is produced by firing raw materials such as limestone, marl, and clay in a rotary kiln at 1450 degrees Celsius. The resulting sinter is then milled with lime sulfate (gypsum), which acts as a setting time regulator. During the grinding of the sinter, so-called non-clinker components, namely granulated blast furnace slag, fly ash, and limestone may be added to act as a filler. One of the important features of cement is hydration, that is, the totality of physical and chemical processes (including dissolution, hydration, and hydrolysis reactions) due to the combination of water and cement with the formation of reaction products.

Cementitious composites include the following components or various combinations of them: cement, fine aggregates and/or coarse aggregates, sand, water, and various additives and admixtures, including plasticizers, superplasticizers, macro, micro, and nano components, as well as various materials are used to modify the parameters of cement composites such as straw, wood chips, recycled rubber. Each added ingredient can improve but also deteriorate the material’s properties. Looking at the phenomenon of hydration occurring in cement composites, the cement paste changes more during this process than the aggregate under temperature changes. Temperature so affects the cement paste that it causes it to shrink during the binding reactions that occur. Note that if the material changes its volume in a bounded space, this can cause stresses to occur in the material. If the contraction and expansion of the material occur in a limited space, the stresses can cause the material to exceed its strength limit, which will cause cracks to appear in the material’s structure.

It is not only the hydration process that can affect the deterioration of the properties and strength of cementitious composites. Other factors include exposure of the material to freeze-thaw cycles, among others, or water penetration into the internal structure, which can transport hazardous substances such as water-soluble salts [23,24,25,26,27,28,29,30,31]. Therefore, scientists worldwide are studying various materials that would help extend the life of cement composites [32]. One such material is nanocellulose, the addition of which can affect strength and durability parameters starting in the nano zone.

Referring to the high specific surface area of nanocellulose leads to improved adhesion between cement matrix particles [33]. In addition, the formation of hydrogen bonds between the nanocellulose and the cement matrix is facilitated by the increased number of hydroxyl groups available on the cellulose. Also, the curing process itself can positively influence the performance of nanocellulose reinforcements in the cement matrix. After accelerated carbonation curing, the porous structure is refined, thereby reducing the alkalinity of the cement matrix. What follows is a better adhesive interface between the cement matrix particles and nanofibers. Consequently, there is better stress distribution, thickening of the cement matrix, and improvement of the mechanical properties of cement composites [34]. In Ref. [35], the authors showed that the modulus of rupture is strongly related both to the interface between the fibers and the matrix and to the performance of the fibers used as reinforcement. It shows that nanocellulose contributes to improving stress transfer alongside the specimen volume when subjected to loading. Sun et al. [36] in their work showed that by adding nanocellulose, a channel is formed between the unreacted core of the cement particle and the pore solution through the C-S-H coating. As a result, the degree of hydration is increased due to the addition of nanocellulose.

## 3. Cellulose and Nanocellulose

**Figure 1:**
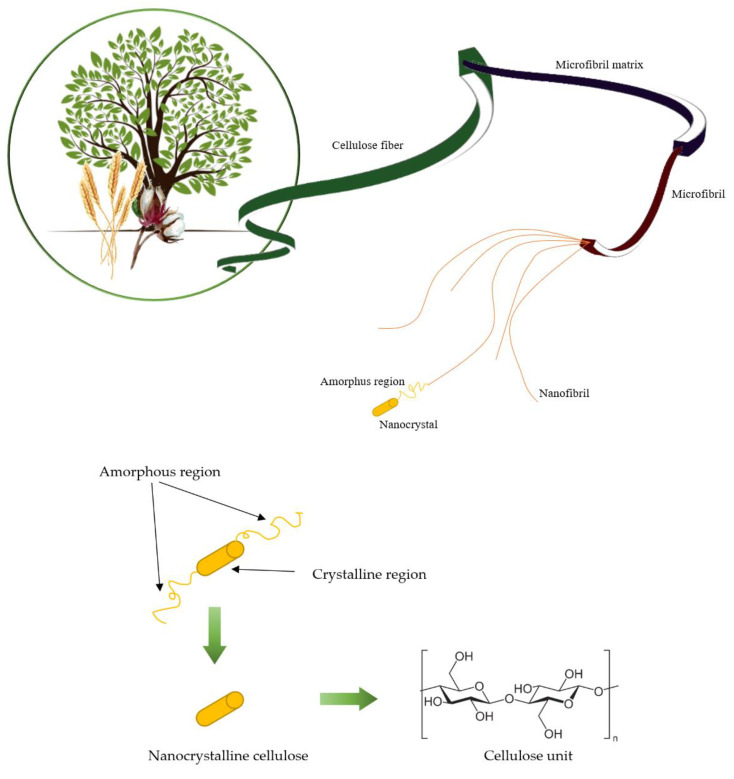
From cellulose fibers to nanocellulose.

In recent years, researchers have focused more and more on using renewable, biodegradable, and environmentally friendly raw materials, including cellulose waste, which can significantly improve cementitious materials’ properties while simultaneously fulfilling sustainable development goals. Due to its strength properties, lightness, renewability, abundance, and environmental friendliness, cellulose has been widely used in construction. Cellulose fibers (Figure 1) are commonly available from wood or annual plants and are materials for reinforcing matrices such as polymer or fiber—cement composites, as evidenced by a significant number of recently published reviews and special issues (Table 1). The development of nanomaterials in recent years has turned the attention of researchers toward nanomaterials and nanocellulose [37,38,39,40].

Cellulose is nature’s most abundant biopolymer and a significant component of the plant cell wall. Besides plants, cellulose can be synthesized by bacteria or found in algae and tunicates [39,55]. Chemically, cellulose consists of D-glucopyranose units linked by β-1,4-glycosidic bonds, with a repeating cellobiose unit organized into fibers. Cellulose can be found in seven allomorphs (cellulose I_α_, I_β_, II, III_I_, III_II_, IV_I_, and IV_II_), of which cellulose I is the most crystalline in structure. Cellulose I_β_ is more thermally stable than cellulose I_α_ because there are weaker hydrogen bonds in cellulose I_α_ than in cellulose I_β_.

Single cellulose fibers are known for their unique properties, such as high mechanical strength, which puts them in the same league as Kevlar fibers or steel wire [50,55,56,57]. Due to cellulose’s linear and quite regular structure and many hydroxyl groups in the molecule, cellulose polymers can form ordered crystalline structures and amorphous regions with disordered structures. The crystalline regions give cellulose fibers critical mechanical properties. Hydroxyl groups in a cellulose polymer can form hydrogen bonds within the polymer itself (intramolecular hydrogen bonds) or between adjacent cellulose polymers (intermolecular hydrogen bonds). Intramolecular hydrogen bonds are responsible for the stiffness of cellulose polymer chains, while intermolecular hydrogen bonds allow linear polymers to form sheet structures.

Furthermore, the amphiphilic nature of cellulose results from the equatorial arrangement of the hydroxyl groups (hydrophilic nature) at the ring and the hydrogen atoms of the C-H bonds (hydrophobic nature), which are located at the axial positions of the ring. Highly significant effects on interactions and properties, such as solubility of cellulose structures, are expected. Overall, hydrogen bonding networks are firm and tightly packed in the crystalline parts of cellulose fibers, leading to a tough, strong, fibrous, water-insoluble, and highly resistant to most organic solvents plant cell wall. Thanks to many hydroxyl groups, cellulose particles can absorb water when mixed with cementitious materials and act as supporting or reinforcing fillers in many polymer systems [50,55,58].

Nanocellulose can be defined as a cellulosic material with at least one dimension on the nanometer scale (1 nm = 10 × 10^−9^ m) [40]. Typically, nanocellulose is obtained from cellulose through a series of chemical or physical treatments. Nano-sized cellulose materials are usually prepared from extracted cellulose or highly refined cellulose products such as wood pulp, rice straw, commercial cotton and industrial waste cotton, and processed fruit and vegetable waste. In addition, nanocellulose also be synthesized by algae, tunicate, and bacteria [50,57]. Nanocellulose can be chemically functionalized to meet specific needs by modifying the hydroxyl groups and tailoring the degree of hydrophilicity [38]. The structural, physicochemical, mechanical, and biological properties of nanocellulose are highly dependent on its source, synthesis methods, and pre- and post-synthesis processing conditions, which consequently results in various applications of nanocellulose in different fields [39]. Most commonly, nanocellulose is classified as cellulose nanocrystals (CNC), cellulose nanofibers (CNF), and bacterial nanocellulose (BNC) [40]. While all types of nanocellulose are similar in chemical composition, they differ in morphology, particle size, crystallinity, and some properties due to differences in sources and extraction methods [40]. CNCs and CNFs are the best known and are usually used as additives to modify the properties of cement composites [57,59]. However, there are also reports in the literature about lesser-known forms of nanocellulose, that is, spherical (SNC) and square and rectangular-shaped nanocellulose (SSNC and RNC) [60,61]. Different types of nanocellulose differ depending on the source of raw materials, synthesis methods, and structural characteristics. The characteristics of each type of nanocellulose are explored below and in Table 2.

Cellulose nanocrystals (CNC) are known as nanocrystalline cellulose, cellulose whiskers, or cellulose nanowhiskers. CNCs are high-strength nanocellulose typically extracted from cellulose fibers by acid hydrolysis. Acid treatment can remove most amorphous cellulose and produce high-purity cellulose crystals, so CNCs have high crystallinity. CNC contains 64–98% cellulose I_β_, depending on the source of origin, with nearly perfect crystallinity (up to 90%). Morphologically, the CNCs exist in short rods-like shape or whisker shape structures. CNC length and diameter typically range from a length of 200–500 nm to a diameter of 3–35 nm [55,56]. CNCs have unique properties such as high crystallinity, relatively high aspect ratio (10–100), high thermal stability (up to 300 °C), low density (~1.6 g/cm^3^), low coefficient of thermal expansion, large surface area, high tensile strength, and high tensile modulus (up to 170 GPa). Moreover, CNCs, due to the accessible hydroxyl groups on their surface, can be easily functionalized [38,50].

Cellulose nanofibrils (CNF) are also known as nanofibril cellulose, nanofibrillar cellulose, cellulose nanofiber, cellulose microfibril, or microfibrillated cellulose. CNFs are long, flexible, and entangled nanocelluloses that can be separated from cellulose fibers by a mechanical process or a combination of chemical and mechanical treatments. Typically, CNFs are long entangled fibrils with 5–50 nm in diameter and a few micrometers in length [56]. In contrast to CNCs, CNFs contain both amorphous and crystalline domains of cellulose within individual fibers. CNF entanglement may increase the possibility of fiber agglomeration compared to CNC. It is characterized by a high aspect ratio (length to diameter), low density, and large specific surface area, which enables functionalization. There are many hydroxyl groups in CNF, which are more readily available for surface modification than nanocrystalline cellulose (CNC) [50,55].

Bacterial nanocellulose (BNC) is also known as microbial cellulose. It is typically produced by bacterial or microbial species that build low molecular weight sugars (primarily *Gluconacetobacter xylinus*, *Rhizobium*, *Agrobacterium*, *Pseudomonas*, and *Acetobacter*, etc.) within a few days up to two weeks and does not require additional processing to remove contaminants such as lignin, pectin, hemicelluloses and so on [55,56,58,62]. Bacterial nanocellulose has a similar chemical composition to CNC and CNF, but BNC is in the form of twisted ribbons with diameters ranging from 20–100 nm and micrometer lengths, with a large surface area per unit. The morphology of the BNC can vary depending on the type of bacterial strain, the culture conditions, and the bioreactor used [56,62].

Spherical nanocellulose (SNC) is shaped like a sphere with an average size of about 30–50 nm, usually with excellent monodispersity and uniformity. This type of nanocellulose is little known in the literature and less studied than CNC, CNF, and BNC. However, many works are related to the preparation and characterization of spherical nanocellulose [61,63,64,65]. Unlike CNC, CNF, and BNC, SNC can be obtained both by chemical treatment of cellulose source or produced by microorganisms. In addition, spherical cellulose is predominantly cellulose II with a polymorphic crystal structure and relatively uniform particle size.

Rectangular and square-shaped nanocellulose (RNC/SSNC) are other lesser-known types of nanocellulose. The approximate diameter ranges for RNC and SSNC are 30 to 60 nm and 10 to 90 nm, respectively. They are most often obtained by chemical treatment [60,66]. Particle size and shape affect the properties of nanocellulose, which largely determines its applications. CNCs and CNFs are excellent agents for improving the mechanical properties of cement composites, while nanocellulose with a spherical or square structure is an excellent candidate as an emulsion stabilizer or drug carrier for encapsulation [57,59,66].

**Table 2:** Characterization of nanocelluloses in terms of types, source materials, synthesis methods, and critical features.

Type	Source	Synthesis Method	Features	Ref.
Cellulose nanocrystals (CNC)	cotton linters	single-step ammonium persulfate-assisted swelling, followed by oxidation	the high crystallinity index of 90.5% thermally stable excellent dispersibility	[67]
	commercial microcrystalline cellulose	facile and rapid one-step hydrolysis by H_2_SO_4_/HNO_3_ mixed acid	high aspect ratio	[68]
	lignocellulosic biomass	hydrolysis by Ni(II)-transition metal salt followed by washing with distilled water, centrifugation, sonication, and dialysis	crystallinity: 78.8–90.5% diameter less than 100 nm (ranging from 8.8 to 67.8 nm) thermally stable	[69]
Cellulose nanofibril (CNF)	banana peel	alkaline treatment, bleaching, and acid hydrolysis, and alkaline treatment and hydrolysis with xylanase	higher aspect ratio more stable suspension	[70]
	sugarcane bagasse	set of recombinant enzymes: endoglucanase, xylanase and a lytic polysaccharide monooxygenase	much longer and more thermostable compared to the CNF prepared by TEMPO-mediated oxidation	[71]
	raw spruce cellulose pulp and α-cellulose	N-methylmorpholine-N-oxide method	diameters ˂ 500 nm	[72]
Bacterial nanocellulose (BNC)	Bacterial strain Komagataeibacter xylinus (BCC529)	Static culture for 96 h at 30 °C	uniform in a film shape 20–40 nm resistant to high temperatures and good flame retardancy	[73]
Spherical nanocellulose (SNC)	oil palm empty fruit bunch pulp	ultrasound-assisted hydrolysis	high cellulose content—87.7%	[74]
Rectangular and square-shaped nanocellulose (RNC/SSNC)	walnut shell	2,2,6,6-tetramethylpiperidine-1-oxyl radical (TEMPO) oxidation and sulfuric acid hydrolysis	rectangular shape with a length of 55–82 nm and a width of 49–81 nm crystallinity 40.1% thermally stable	[66]

## 4. Methodology for Preparation of Nano-Sized Cellulose

Over the years, different processes have been used to obtain highly purified nanocellulose based on its source and final application. These methods include mechanical, chemical, and biological treatments depending on the pretreatments required. The preparation of plant-derived nanocellulose typically includes mechanical destruction such as high-pressure homogenization, grinding, sonication, and chemical treatment with various acids or a combination of both. The acid hydrolysis process produces a tremendous amount of wastewater containing acid, the mechanical methods utilize too much energy, and the oxidation and ionic liquid methods require costly reagents. Furthermore, enzymatic digestion is also used to prepare wood-based nanocellulose. Therefore, in addition to further solving the existing obstacles in a particular method, it is also necessary to search for new ways to obtain nanosized cellulose and perhaps new types of nanocellulose [62,63]. As mentioned in the previous section, the geometric properties of nanocellulose structures (shape, length, and diameter) mainly depend on the origin of the cellulose and the extraction process and influence the properties and final applications of nano-sized cellulose [39,55,56].

The following section provides an overview of methods for isolating nanocellulose from various sources. Different sources, types, sizes, and processing methods of nanocellulose are shown in Table 2.

### 4.1. Mechanical Processing

Mechanical pre-treatment of cellulose fibers is used to produce fine fibers and includes various isolation processes such as high-pressure homogenization, sonication, grinding and ball-milling, and cryo-crushing methods [39,62]. Besides fragmentation, pre-treatment of cellulose fibers is essential to reduce energy and chemical reagent consumption in cellulose nanofibrillation processes. Moreover, it improves the fibrillation process as the cellulose fibers are isolated by applying a high shear force that splits the cellulose fibers in the longitudinal axis, resulting in the formation of nanofibrillated cellulose. In addition, mechanical treatment can be used independently but is usually combined with other pretreatment methods to reduce energy consumption and increase the yield of obtaining nanocellulose [56].

One of the numerous methods for refining cellulose fibers is high-pressure homogenization (HPH), which was implemented for the first time in 1983 to utilize cellulose nanofibers produced from wood pulp. High-pressure homogenization (HPH) is carried out by passing cellulose slurry into a tank with high pressure and high velocity. The impact and shear force in the liquid cause the cellulose fibers to split into micro- and nano-meter sizes. Numerous authors have reported a reduction in the crystallinity of nanocellulose compared to the original cellulose due to the disruption of intermolecular and intramolecular hydrogen bonds in cellulose during the high-pressure homogenization process [55,75]. This method is most commonly used to produce nanocellulose from raw materials such as wood pulp, bleached sugar beet, prickly pear extract, and kenaf bast fiber [39]. Ultrasonication is the process of isolation of cellulose fibers by the hydrodynamic forces of ultrasound. This method generates a mechanical oscillatory force that causes the formation, expansion, and implosion of microscopic gas bubbles when the liquid molecules absorb the ultrasound energy. Small fiber sizes can be obtained by chopping cellulosic fibers before ultrasonication. Sonication is usually performed after chemical pretreatment of natural fibers to isolate nanocellulose. High-pressure homogenization and ultrasound can also be used to further improve fibrillation [39]. Ball milling is one of the mechanical processing methods that can cause effective defibrillation of cellulose fibers. Due to the centrifugal force of the rotor, shear forces are generated among balls and between the balls and the pot’s surface. As a result, cellulose fibers are crushed into smaller diameter sizes. It is worth noting that ball milling shows excellent potential for further development in nanocellulose extraction and application [55]. Production of nanocellulose by ball milling is greatly influenced by ball size, ball, and material weight ratio, milling time, and moisture content. Even though this process is very efficient in preparing nanocellulose fibers, homogeneity is still a significant issue [39]. Cryocrushing separates nanocellulose in which cellulose fibers are treated with liquid nitrogen to produce ice crystals that exert more pressure on the cell wall, leading to cell wall decomposition and CNF formation [62]. Solidified cellulose raw material is crushed using a mortar and pestle to transform cellulose into nano-sized fibers [39].

### 4.2. Chemical Processing

Chemical methods for isolating nanocellulose from cellulosic materials include alkaline pretreatment, acid pretreatment, acid hydrolysis, oxidation, ionic solvent treatment, and various solvent isolation methods. Alkali-acid pretreatment is the most common method to dissolve lignin, hemicellulose, and pectin before mechanical isolation or acid hydrolysis [55].

Alkali-acid pretreatment is most widely performed to dissolve lignin, hemicellulose, and pectin before mechanical isolation of NFCs. Acid pretreatment, also known as acid-chlorite treatment or bleaching process. This process dissolves hemicellulose, most lignin, and other components from cellulosic materials. The chemical reagents most commonly used in this process are sodium chlorate, acetic acid, and hydrochloric acid. After this step, the white color of the holocellulose fibers indicates the successful removal of lignin and other impurities [56]. Alkaline pretreatment effectively increases cellulose yield and removes lignin (disrupts lignin structure and breaks the bonds between cellulose and lignin). The usual alkali is sodium hydroxide (1–20 wt.%), which is mixed with cellulose for 1–5 h. Then, the obtained solid products are washed with distilled water to reach a neutral pH and dried in an oven at 50 °C. The fiber products obtained from this treatment are mainly in the form of cellulose, and other non-cellulosic materials were removed. Cellulose fibers require careful alkaline treatment to avoid unwanted cellulose degradation so that in the subsequent step, acid hydrolysis is carried out only on the fiber surface [39,55].

Acid hydrolysis is the oldest, most widely and routinely used method for obtaining nanocellulose (CNC) from cellulosic raw materials. Because of the combination of ordered and disordered regions in cellulose chains, disordered regions can be easily hydrolyzed by acid, and ordered components remain residues [55]. Hydrogen ions from the acid molecules penetrate the amorphous regions of cellulose chains and facilitate the hydrolytic breakdown of glycosidic bonds. In recent years, various strong acids have been used to degrade cellulose fibers, but sulfuric acid is the most commonly used. Phosphoric acid, hydrochloric acid, hydrobromic acid, and nitric acid are also recommended to prepare crystalline cellulose nanoparticles [56,62].

The advantage of using sulfuric acid as a hydrolyzing agent is that it initiates the esterification process on the cellulose surface and promotes the grafting of anionic sulfate ester groups. Moreover, the presence of anionic groups induces the formation of a negative electrostatic layer on the surface of nanocrystals and facilitates their dispersion in water. Thus, acid hydrolysis can not only strongly isolate nanocrystalline cellulose but also make nanocellulose dispersed as a stable colloidal system due to sulfate ions’ esterification of the hydroxyl group. However, this decreases the thermostability of CNC nanoparticles, which can be improved by neutralizing CNC with sodium hydroxide. However, nanoparticles obtained by acid hydrolysis have a high aspect ratio and are rod-shaped. Their geometric dimensions depend on the cellulose source and hydrolysis method [56]. The main parameters affecting acid hydrolysis are acid concentration, hydrolysis reaction temperature, and cellulosic waste-to-acid volume ratio. The main drawback of acid hydrolysis is the toxic and corrosive nature of concentrated acids [62] and acidic wastewater generated during the washing process to neutralize the pH value of the nanocellulose suspension. The washing process is usually carried out either by adding cold water followed by centrifugation until a neutral pH is reached or using an alkaline substance, such as sodium hydroxide solution, to neutralize the pH value.

Recently, other methods have been studied that degrade the amorphous domain from cellulosic fibers, such as oxidation and treatment with Ionic liquids (ILs). Oxidative pretreatment, such as the oxidative process by TEMPO (2,2,6,6-tetramethylpiperidine-1-oxyl radical), proceeds on the surface of microfibrils and creates a negative charge that causes nanofiber repulsion and fibrillation. Oxidative pretreatment solves the aggregation issue caused by the presence of-OH groups and introduces new functional groups, such as carboxyl (-COOH) and aldehyde (-COH) groups, to the cellulose surface under aqueous and mildly acidic conditions [39]. TEMPO-oxidized cellulose nanofibers (ONC) are consistently uniform in width (3–4 nm) and have a high aspect ratio, making them suitable for use as transparent and flexible displays, gas-tight films for packaging, and filler nanofibers for composite materials [55]. Ionic liquids (ILs) are organic salts with specific properties such as non-flammability, thermal and chemical stability, and infinitely low vapor pressure. Recently, ILs have received increasing interest from researchers as solvents for cellulosic materials [56]. However, this pretreatment route has serious drawbacks, such as the high cost of organic solvents and instrumentation, and the volatile organic solvents limit its industrial application [62].

### 4.3. Enzymatic Processes

Enzymatic hydrolysis is a biological treatment process in which enzymes are utilized to digest or modify cellulosic fibers. Enzymatic pretreatment of cellulose is sometimes considered a “green method” because it does not require the use of chemical reagents. Enzymes with selective hydrolysis ability can degrade or modify the lignin and hemicellulose content without disturbing the cellulose content. Because cellulose fibers are composed of various organic compounds, one specific enzyme cannot destroy the fiber. A set of enzymes, collectively called cellobiohydrolases and endoglucanases, is required to break down cellulose by enzymatic hydrolysis. Cellobiohydrolases (cellulose types A and B) are directly breaking down highly crystalline cellulose, while endoglucanases (C- and D-type cellulases) target and break down the disordered structure of cellulose. These two cellulose-degrading enzymes exhibit a synergistic effect during cellulose fibrillation [39,56]. Generally, biological treatment with enzymes can be carried out under mild conditions but requires a long operation time. To address this issue, enzymatic hydrolysis is usually combined with other methods, such as ionic liquids. Enzymatic pretreatment has many advantages: high yield, environmentally friendly, high selectivity, minimal energy cost, and milder reaction conditions than other chemical processes. The main drawbacks of this method include higher enzyme cost and longer processing time required to disintegrate cellulose compared to other methods [62].

### 4.4. Bacterial Synthesis

Bacterial nanocellulose (BNC) is obtained using bacteria (i.e., *Acetobacter*, *Rhizobium*, *Agrobacterium*, *Aerobacter*, *Achromobacter*, *Azotobacter*, *Salmonella*, *Escherichia*) and produced by utilizing glucose and other carbon sources. Bacterial nanocellulose is produced using a ‘bottom up’ method by different strains of microorganisms and is the purest form of cellulose. It is synthesized in the form of β-1,4-glucan chains in bacterial cells, which stick out as pro-fibers through terminal complexes found in the cell wall of bacterial cells and crystallize to form ribbon-shaped microfibers and eventually form stacks consisting of bundles. Currently, research on BNCs is mainly focused on their low-cost production, searching for and genetically engineering new bacterial strains to increase productivity, improving existing structural characteristics, and imparting additional features by creating composites with different functional materials [39].

## 5. Properties of Nanocellulose

**Figure 2:**
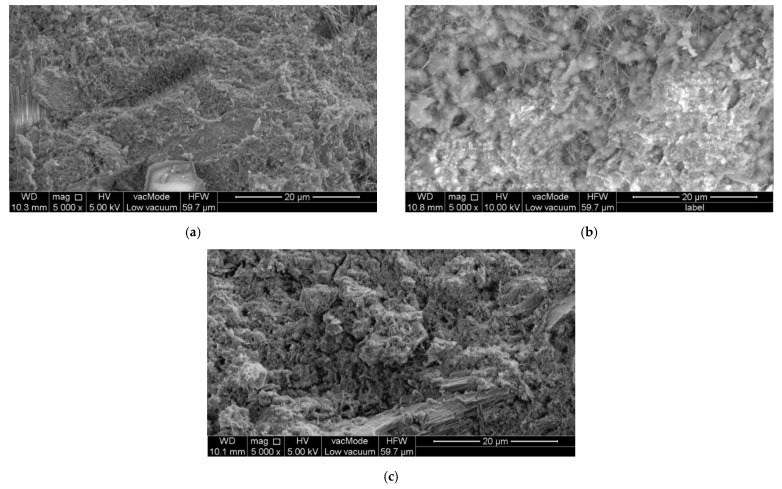
Images taken with a scanning electron microscope (magnification 5000×): (**a**) concrete without NC, (**b**) concrete with CNC, and (**c**) concrete with CNF (own research).

As with other materials, additives, or admixtures added to cementitious composites, it is important to know their properties before using them. It is so important that at a later stage of analysis, knowing the characteristics of the material used, an analysis can be made of how a particular characteristic may have affected the properties of the finished material. Undeniably, one crucial piece of information that should be learned before using the chosen nanocellulose is its dispersion properties [76,77,78,79,80]. It is essential to determine how the nanocellulose will distribute in the material. The worst case is when clusters of nanocellulose particles are formed in one place instead of being distributed evenly throughout the composite. Such a problem also occurs in the case of other fibers, such as steel fibers which, unevenly distributed in the mixture, can cause the formation of so-called ‘hedgehogs’ in the material’s structure. Figure 2 compares the microstructures of concrete without nanocellulose, CNC, and CNF, and Table 3 summarizes the different properties of nanocelluloses.

## 6. Nanocellulose Surface Modifications

**Figure 3:**
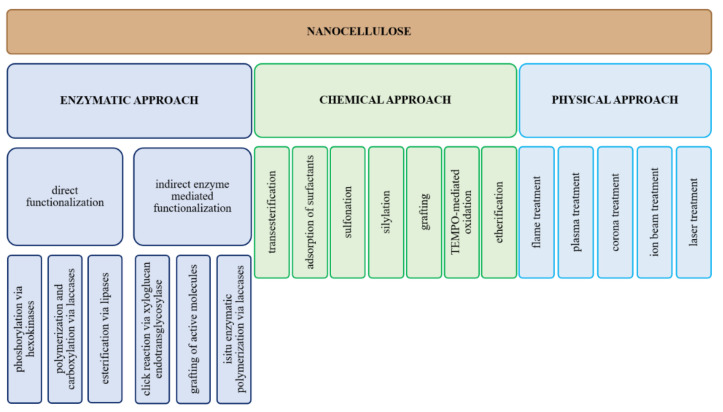
Approaches to surface modification of nanocellulose.

Some scientists, in their research, are subjecting nanocellulose to surface modification to improve the properties of nanocellulose and obtain better properties of the final material [81,82,83,84]. There are various surface modification methods of nanocellulose [85,86,87,88,89,90,91,92,93,94]. Figure 3 shows the most common approaches to surface modification of nanocellulose.

**Table 3:** Characterization of nanocelluloses in terms of their properties.

Type	Source	Preparation Method	Diameter	Length	Width	Shape	Degradation Temperature	Crystallinity Index	Zeta Potential	Ref.
			nm	Nm	nm		T_max,_ °C	CrI, %	mV	
CNC	cotton	H_3_PO_4_ hydrolysis	31 ± 14	−	−	rod-like	−	−	−	[95]
	commercial microcrystalline cellulose	H_2_SO_4_/HNO_3_ mixed acid hydrolysis 50 °C	−	223 ± 16	14 ± 5	rod-like	318.6	89.8	−35.0	[68]
SNC	cotton clothes (100% cotton)	H_2_SO_4_ acid hydrolysis	14 ± 4	−	−	spherical	351	95	−46.8 ± 1.6	[96]
	cotton linter	heterogeneous acid-catalyzed hydrolysis (Amberlite IR 120)	25–45	−	−	spherical or corn-like	391	84	-	[97]
	baby diaper waste	H_2_SO_4_ acid hydrolysis	10–20	−	−	spherical	−	65.1	−5.84	[98]
CNF	corn husk	high-intensity ultrasonication	20.14 ± 4.32	−	−	slender interconnected webs	348	53.4	−	[99]
	ushar seed fiber	TEMPO-oxidation	10–20	−	−	web-like	316	59	−	[100]
	corncob residue	PFI refining	43.1 ± 25.3	−	−	twisted structure	305	49.9	−23.1 ± 2.3	[101]
BNC	grape pomace extract and corn steep liquor	the bacterial strain of *G. xylinus* NRRL B-42	−	−	18–57	ribbon	350 ± 4	68 (21 d) 85 (30 d)	−	[102]
	pineapple and watermelon peels	bacterial culture *Komagataeibacter hansenii*	−	−	70–130	flat twisted ribbonlike fibrils	−	67	−	[103]
RNC/SSNC	China cotton (CC), south African cotton (SAC), waste tissue papers (TP)	acid hydrolysis	10–90	−	−	rectangular and square	230–250	89.9–97.8	−	[60]
	walnut shell	2,2,6,6-tetramethylpiperidine-1-oxyl radical oxidation	−	55–82	49–81	rectangular	250	40.1	−	[66]

## 7. Modification Methods of Cement Composites with Nanocellulose and Surface Modified Nanocellulose and Effect of Nanocellulose Types on Physical, Chemical and Mechanical Properties of Cement Composites

### 7.1. Akhlaghi et al.: Application of Bacterial Nanocellulose Fibers as Reinforcement in Cement Composites [83]

Nanocellulose: bacterial nanocellulose (BNC) produced by *Gluconacetobacter Xylinus* bacteria.

Cement composite type: mortar.

w/c ratio: 0.5.

Use: direct-powder and gel added to cement mix; indirect-BC coated onto the polypropylene fibers.

NC dosage: direct—0.1%, 0.3%, and 0.5% by volume of cement; indirect—0.5%, 1.0% and, 1.5% by volume of mortar.

Procedure for adding NC: direct—BNC added to batch water; indirect—polypropylene fibers treatment with BNCs added to mortar mix.

Components of the mixtures: sand, Ordinary Portland cement (GEM I—32.5), water, polypropylene fibers coated with BNC, polypropylene fibers, BNC gel, BNC powder, superplasticizer.

Results:

- flexural strength—(direct) the best results were achieved after adding 0.5 by volume of cement BNC powder. It may be because the powder filled the nanopores more and thus contributed to the final value. Compared to the reference mortar, the strength increased by 104%. Excellent results were also achieved with the use of 0.3% BNC powder, the strength increased by 94% compared to the reference mortar; (indirect) in all cases there was a reduction in strength by 20, 23, and 40% (unmodified fibers) and 17, 8, and 5% (modified fibers) compared to the reference mortar which could be caused by the uneven distribution of fibers in the structure of the material.
- compressive strength—the addition of 0.3% BNC gel contributed to the most significant increase in strength by 22% compared to control samples. Looking at the BNC content percentages, the strength improved in 5 out of 6 cases, but the addition of 0.5% BNC gel resulted in an 8% decrease in strength compared to the reference sample. It is possible that this was caused by the adhesive forces of the BNC gel, and the gel was not distributed evenly in the material structure; (indirect) plain fibers and modified fibers have contributed to a decrease in strength in all cases. Samples reached the highest decrease, equal to 15%, with the addition of 1.5% plain fibers. However, modifying the fibers with BNCs resulted in strength increases of 4, 8, and 14% in each of the cases where this modification was used (0.5, 1, and 1.5%), compared to the same doses of plain fibers. It may be due to the greater contact surface area with the cement matrix.
- water absorption—(direct) all dosages of BNC powder and gel decreased mortar saturation compared to the reference material. The decrease was from 37 to 6%, where the addition of BNC powder at 0.3% contributed to the 37% decrease. The BNC powder and gel sealed the structure of the mortars; (indirect) ordinary fibers caused an increase in water absorption by 24, 46, and 83% compared to reference samples. Modified fibers also affected this parameter in this way, but at the same caused a decrease in water absorption by 14, 17, and 25% (0.5, 1, and 1.5%) compared to plain fibers. The addition of fibers increased the absorbency because the material’s porosity increased due to their addition.

### 7.2. Diamanti et al.: Suspended Multifunctional Nanocellulose as Additive for Mortars [104]

Nanocellulose: Oxidized nanocellulose (ONC) from cotton wool.

Cement composite type: mortar.

w/c ratio: 0.48.

Use: direct.

NC dosage: 0.3%, 0.6%, 1.2%, and 2.4% by weight of cement.

Procedure for adding NC: suspension of the ONCs added to the premixed commercial mortar. CrI about 65%.

Components of the mixtures: Portland cement—based premixed commercial mortar Webercem RA30 by Weber, water, ONC.

Results:

- compressive strength—the addition of 2.4% ONC contributed to the most significant increase in strength by 34% compared to control samples. Also, the addition of 1.2% ONC increased the strength, at values of 0.3% and 0.6% showing no significant increase in strength and even a decrease in strength after the addition of 0.3% ONC compared to control samples. The higher percentage addition of ONC helped reduce the material’s porosity and thus increase strength.
- water absorption—as in the case of strength, ONC content affected water absorption. That is, samples with 2.4% ONC content had the lowest absorption compared to other samples and reference samples. The addition of 0.3% ONC increased the water absorption, meaning that this percentage negatively affected the material’s microstructure, just as it did for compressive strength.
- porosity tests—as in the case of strength, ONC content affected water absorption. That is, samples with 2.4% ONC content had the lowest. The results of the study presented by the authors showed that at ONC contents of 0.0%, 0.3%, and 0.6%, the content of large capillary pores is the most prominent—pores above 1μm, whereas in the case of ONC contents of 1.2% and 2.4% there were small and large capillary pores with diameters from 10 nm–1μm. Large capillary pores cause rapid movement of free water due to capillary forces. Therefore, the higher ONC content positively affected the characteristics mentioned above.
- thermal gravimetric analyses—thermal gravimetric studies have shown that the presence of ONC has no effect on the material’s thermal stability.

### 7.3. Nasir et al.: Engineered Cellulose Nanocrystals—Based Cement Mortar from Office Paper Waste: Flow, Strength, Microstructure, and Thermal Properties [105]

Nanocellulose: cellulose nanocrystals (CNC) from office paper waste.

Cement composite type: mortar.

w/c ratio: 0.50.

Use: direct.

NC dosage: 0.25%, 0.5%, 0.75%, 1.0%, and 1.5% by weight of cement.

Procedure for adding NC: powder—three different CNCs added as an additive to cement mixes. NC was sonicated with water before being added to the cement mix. Cement and water with NC were added to the mixer. The sand was then added. The mixture was stirred at low speed while adding the ingredients. Only after all the ingredients were mixed was the mixer set to high speed and mixed for 60 s. The CNCs had different crystallinity, diameter, and length and were synthesized in different solid-to-acid ratios (CNCs designations: C1, C2, C3 for which the crystallinity index (CrI) was 79.91%, 84.23%, and 89.31%, respectively).

Components of the mixtures: type-I ordinary Portland cement, sand, water, CNC.

Results:

- flow—all doses of nanocellulose caused a decrease in flow diameter. The flow diameter for the mortar without CNC was the largest, and each dose gradually reduced its diameter regardless of the type of CNC. The decrease in each case was linear due to a larger CNC area and, thus, increased water demand. Such a trend may affect the workability and compaction of the material. Other authors also noted this behavior, among others [52,106]. The most significant percentage decrease in the size of the flow diameter was recorded for CNC labeled C2 at a dose of 1.5% and was 42% and the smallest for C1 at a dose of 0.25%, amounting to 5%, compared to the reference mortar.
- compressive strength—compressive strength testing was conducted at 3 intervals after 7, 14, and 28 days. Comparing the strengths after 28 days, it can be seen that the greatest increase was recorded for C1 mortar, for doses of 0.75%, 1.0%, and 1.5%. However, the authors pointed out that from an economic point of view, it is not economically viable to use doses of 1.0% and 1.5% for C1 mortar because the difference in strength compared to the 0.75% dose is insignificant. Therefore, they indicated that the best strength should be considered the strength after 28 days for the 0.75% dose for C1, which increased the strength by 21.9% over the reference mortar. For C2, an increase in strength was noted for the 0.75% dose and for C3 for the 0.25%, 0.5%, and 1.0% doses. In each case, the increase in strength was linear. There was no situation where, for example, after 3 days, there was higher strength than after 7. The optimal dose for CNC: C1, C2, and C3, a dose of 0.75%, was considered.
- flexural strength—strength, as in the case of compressive strength, was tested after 3, 7, and 28 days. In this case, the increase in strength was also linear. Also, as in the case of compressive strength, the best results were achieved by C1 mortar after 28 days for doses of 0.75%, 1.0%, and 1.5%. The authors found the 0.75% dosage optimal and economical in each case, but the 0.75% dosage for C1 resulted in the most significant strength increase of 31.3% compared to mortar without CNC.
- the volume of permeable voids—identical results to those for strength were obtained for the volume of permeable voids, that is, for mortar C1 for doses of 0.75%, 1.0%, and 1.5%, there was the most significant decrease in the content of permeable voids. It is confirmed by the results obtained for compressive and flexural strengths. The most significant decrease was achieved by C1 mortar for doses of 0.75% and a 1.0% decrease of 14.6%. In the case of this mortar, the decrease in the volume of permeable voids was also linear. In the case of C1 and C2 mortars, the distribution was more parabolic, and the smallest value was reached in the case of the dose of 0.75%. The C1 mortar was denser and thus settled better strength parameters than C2 and C3 mortars.
- thermal conductivity—The highest thermal conductivity of 0.96 W/mK was achieved by the samples for C1 mortar at a dosage of 1.5% CNC—a 13.5% increase in conductivity over the reference mortar. It was the most significant increase of all the mortars and each dose. The C1 mortar, for each dose, achieved higher results than the C2 and C3 mortars. It may be explained by the fact that CNC causes a slowdown in the water loss in the structure, and the lower pore occupancy causes a reduction in air content and, thus, an increase in thermal conductivity.
- mineralogy—after performing the test with FTIR, the authors noted that looking at the peaks obtained for samples C1, C2, and C3 and mortar without CNC, the differences between them were almost imperceptible. However, differences in intensity could be seen, for example, for the portlandite peak. Its intensity was lower than for the mortar sample without CNC. It may indicate that a secondary hydration reaction has occurred. The reaction likely took place between the CNC crystals and lime. It involves the formation of more C-S-H gel, resulting in increased durability of the CNC-added material and increased mechanical strength.
- bond characteristics—based on the FTIR study, the authors hypothesized that the addition of CNC contributed to an increase in the number of hydration products or their density. Also, due to the larger crystals in the C1 samples, there may have been a greater degree of reactivity in these samples. Thus, the structure’s crystallinity and the formation of hydration products were regulated. Consequently, this contributed to the increased strength of the C1 samples. It is possible that the increased strength could also be related to the noted increase in the intensity of the C-S-H gel band.
- morphology and elemental analysis—images taken with a scanning electron microscope (SEM) showed that the microstructure became heterogeneous after the addition of CNC. Images of mortars with 0.75% CNC dosage were more compact than in comparison with samples with higher CNC addition. The increase in CNC content in the samples resulted in more cracks and a weak interfacial transition zone. In the case of C1, the dimensional stability of CNC may have resulted in improved strength parameters through better bridging of cracks formed in the structure. EDS analysis showed increased Si/Ca and Si/Al ratios and C1 samples compared to other samples, which indicates that more aluminum and silicon atoms have precipitated. With these, stronger Si-O-T chains are formed.

### 7.4. Barnat-Hunek et al.: Effect of Cellulose Nanofibrils and Nanocrystals on Physical Properties of Concrete [57]

Nanocellulose: cellulose nanocrystals (CNC) from apple cellulose and cellulose nanofibrils (CNF) from carrot cellulose.

Cement composite type: concrete.

w/c ratio: 0.45.

Use: direct.

NC dosage: 0.5% and 1.0% by weight of cement.

Procedure for adding NC: CNC (CrI 80.90%) and CNF (CrI 74.98%) were used as water suspensions. The water used to make the suspension was subtracted from the amount of water in the reference formulation. Nanocellulose was added along with the batching water to the concrete mixture after the dry components of the mix were mixed in a concrete mixer for 2 min.

Components of the mixtures: Portland cement CEM I 32.5R, quartz sand, fine aggregate 2–8 mm, coarse aggregate 8–16 mm, water, water suspensions of CNC and CNF.

Results:

- the specific density and bulk density—in the case of specific density, there is not much change in values between concretes, but in the case of bulk density, the wattage increased when NC was added to the concrete. The most significant increase compared to the reference concrete was recorded for concrete with CNF 1.0% by 11.9, slightly more extensive than for CNC 1.0% concrete, where the increase was 9.0%.
- absorptivity—the water absorption value decreased in both cases, that is, after the addition of CNC or CNF. The study also showed that the value decreased as the amount of CNC or CNF increased. The most significant decrease in water absorption was achieved by samples that contained CNC in an amount of 1.0%. It shows that the addition of CNC in this amount caused a sealing of the material’s structure and thus reduced the water absorption by 64.3% compared to the reference concrete.
- open porosity—the trend shown for absorbability also occurs for open porosity. As the number of nanocellulose increases, the open porosity value decreases in each case. A minor decrease was recorded in the case of absorbability for CNC 1.0% specimens. The authors for CNC 1.0% concrete recorded the most significant decrease in open porosity of 48.8% compared to concrete without NC.
- compressive strength—strength value increased by 37.9% when 1.0% CNC was added to the base mixture. It was the most significant increase comparing all concretes with NC. The rest of the concretes with NC also showed an increase in this value compared to the concrete without NC. The addition of NC did not cause a decrease in compressive strength but rather an increase. This tendency may be due to the shape of the CNC fibers.
- tensile strength—the tremendous increase in strength, in this case, was achieved by concrete with the addition of 1.0% NCF. The strength increased by 34.5% compared to concrete without NC. As in the case of compressive strength, an increase in strength was noted for each type of concrete with NC. The most significant increase for concrete with NCF may be due to the shape of the fibers—longer fibers may have inhibited more propagating cracks in the material.
- porosity (mercury injection capillary pressure method (MICP))—the study showed that a 1.0% CNC admixture reduced Cumulative pore volume the most. The authors noted a 40.7% decrease compared to the reference concrete. In this case, too, all admixtures contributed to the decrease in this value. Not only did the cumulative pore volume value decrease with increasing admixture, but also the dominant pore sizes. The largest dominant pore size was in the reference concrete, equal to 135 nm, but the addition of 1.0% CNC and NCF already contributed to a decrease in this value by 79.2% (28 nm) and 76.3% (32 nm), respectively.
- contact angle (CA) and surface free energy (SFE)—The value of CA increased with increasing NC content in the concrete. Measurements were taken after 0 and 5 min. The highest value after 5 min was reached by the 1.0% CNC concrete of 63°, which was 12.6 times higher than the value for the reference concrete. As well as, the SFE value for the 1.0% CNC concrete was the smallest at 46.1 mJ/m^2^—the highest hydrophobicity for this concrete was achieved (value after 5 min). The SFE value for the reference concrete after 5 min was 72.5 mJ/m^2^.
- freezing-thawing resistance (after 100 F-T cycles)—the above results were reflected in the results related to freezing-thawing resistance. Specimens achieved a minor decrease in compressive strength with 1.0% CNC admixture, where the decrease was only 0.18%. In comparison, for concrete without NC, this decrease was 2.38%.
- morphology and elemental analysis—the analysis showed that all concretes contained calcium, aluminum, and silicon oxides. Compared to all concretes, higher silicon dioxide content was observed in concretes with CNC content. CNC 1.0% concrete is characterized by a more compact structure than other NC concretes. It also has fewer pores in the structure and micro-cracks than concrete without NC. It is reflected in the results of durability tests.

### 7.5. Kamasamudram et al.: Cellulose Nanofibrils with and without Nanosilica for the Performance Enhancement of Portland Cement Systems [107]

Nanocellulose: cellulose nanofibrils (CNF), cellulose nanofibrils modified with nanosilica (Si-CNF)—solid concentration of about 3%. CNF from the Process Development Center (PDC) at the University of Maine is produced from bleached softwood pulp.

Cement composite type: cement paste.

w/c ratio: 0.35 and 0.45.

Use: direct.

NC dosage: 0.025%, 0.05%, 0.1%, 0.3%, and 0.5% by weight of cement.

Procedure for adding NC: CNF or Si-CNF was mixed with the water, cement was added, and all the ingredients were mixed. The water used to make the suspension was subtracted from the amount of water in the reference formulation. CrI of CNF 67–88%.

Components of the mixtures: ordinary Portland cement type I/II, water, CNF slurry, Si-CNF slurry. Tetraethyl orthosilicate and sodium hydroxide have been used to coat CNFs with silica nanoparticles.

Results:

- cement hydration—The CNF was coated with a layer of silica, which formed an additional C-S-H at the interface between the fibrils and the grout matrix through nucleation and pozzolanic reaction. This additional C-S-H can ultimately protect the fibrils from alkali attack and strengthen the bond between the fibrils and the cement matrix. The addition of CNF and Si-CNF at a w/c of 0.35 increased the peak heat flow of cement hydration. With CNF, this peak was shifted to the left, from which it can be inferred that the addition of this NC contributed to accelerating the cement hydration process. This effect unfortunately diminished when the w/c ratio increased. The authors did not notice a specific trend related to the acceleration or retardation of the cement hydration process, looking at the amount or type of NC. The increase in total heat release during hydration indicates that the addition of CNF accelerates the process.
- compressive strength—no significant effect of CNF and modified CNF on the increase in compressive strength after 90 days was found. It may be due to inadequate distribution of NC in the material structure. The addition of 0.1% and above of unmodified CNF resulted in a decrease in the material’s compressive strength. An increase in this parameter after 90 days of curing was noted at a dose of 0.05% CNF and was 24% for w/c 0.35 and 15% for w/c 0.45. In the case of Si-CNF, the highest increase after 90 days was found with the addition of 0.5% and was 22% for w/c 0.35 and 14% for w/c 0.45, compared to reference samples.
- fracture properties—tensile strength increased by 75% and 55% for 0.5% CNF and Si-CNF dosage, respectively. The unmodified CNF helped inhibit crack propagation more than Si-CNF, thereby increasing tensile strength. The addition of 0.5% Si-CNF did not significantly affect the elastic modulus. Its increase compared to the control samples was 15%. Otherwise, in the case of CNF, this increase was as high as about 200% and 250% for doses of 0.025% and 0.5%, respectively (compared to reference samples).

### 7.6. Fan et al.: Experiment and Molecular Dynamics Simulation of Functionalized Cellulose Nanocrystals as Reinforcement in Cement Composites [108]

Nanocellulose: cellulose nanocrystals (CNC) from agricultural products: wood pulp and cotton.

Cement composite type: mortar.

w/c ratio: 0.55.

Use: direct—CNCs: CNC-C-containing carboxyl groups and CNC-S-containing sulfo groups; indirect-film CNC coated onto the polypropylene fibers.

NC dosage: direct—0.01%, 0.05%, 0.1%, 0.3% for CNC-C and CNC-S and 0.5% for CNC-S by weight of cement; indirect—0.3% volume ratio of fiber to mortar.

Procedure for adding NC: direct—CNCs added to mortar in the solution state. The automatic mixer was used to disperse CNCs in the mortar; indirect—polypropylene fibers coated with CNC (PPCNC) were added at the end of the preparation of the mixture. CrI—no data available.

Components of the mixtures: ordinary Portland cement 42.5, sand, water, CNCs, PPCNCs, and polypropylene fibers.

Results:

- fluidity and hydration—the doses of 0.01% and 0.05% CNC-C reduce the fluidity of the mortars compared to the reference mortar, and in the case of doses of 0.1% and 0.3%, this value decreased dramatically. For this reason, these mixtures should increase the amount of water used to prepare mortars by 3% and 5%, respectively. By this treatment, the fluidity of the mortars will not change compared to the reference mortar. CNC-C had a more substantial effect on the fluidity of the mortar than CNC-S. The addition of CNC-C and CNC-S affected the increased C-H content in the material structure and decreased the C2S and C3S content.
- compressive strength—the strength of PPCNC-added samples increased by 11% compared to reference samples. The best results were achieved when CNC-C was used at a dosage of 0.05%—in this case, the compressive strength increased by 22.28% compared to the reference samples. Unfortunately, increasing the dosage above 0.05% results in a decrease in strength increase and even a decrease below the value for the reference samples. In the case of CNC-S, there was weak adsorption with C-S-H, which increased the number of pores in the structure resulting in a decrease in strength.
- flexural strength—the most significant increase in flexural strength was noted for the CNC-C 0.05% samples, for which the strength increased by 23% compared to the reference samples. None of the other samples achieved a higher value.
- SEM analysis—CNC affects the growth of hydrated products, changes the shape and size of hydrated crystals, and affects the compactness of the mortar structure. Polypropylene fibers coated with CNC have dramatically changed their morphology. It is because the hydrophilic CNC adhered very closely to the fibers. As a result, the modified fibers have better roughness and higher specific surface area than unmodified fibers.

### 7.7. El-Feky et al. Nano-Fibrillated Cellulose as a Green Alternative to Carbon Nanotubes in Nano-Reinforced Cement Composites [109]

Nanocellulose: cellulose nanofibrils (CNF) from cotton fibers.

Cement composite type: mortar.

w/c ratio: 0.43.

Use: direct.

NC dosage: 0.02%, 0.04%, 0.06%, and 0.08% by weight of cement.

Procedure for adding NC: CNF was added to 600 mL of mixing water and then subjected to indirect sonication (ultrasonic waves through bath sonicator). First, cement was poured into the mixer, then 600 mL of CNF solution was added. The whole mixture was mixed for 2 min. Later, the rest of the mixing water with the superplasticizer was added and mixed for 2 min. Then sand was added, and everything was mixed for another 2 min. CrI—no data available.

Components of the mixtures: ordinary Portland cement (CEM I/42.5R), sand, water, superplasticizer, carbon nano-tubes (CNT), CNF.

Results:

- compressive strength—there was no positive effect of the presence of CNF on early strength for all content ranges tested. However, late-age compressive strength showed a significant increase for most CNF mixtures. The highest early-age compressive strength was 37.3 MPa for cement replacement by 0.02% CNF, with a reduction of 10.1% compared to the reference mix. After 28 days, the maximum compressive strength obtained for the CNF mixes was 51.4 MPa with 0.04% CNF with a 10.68% improvement over the reference mix.
- flexural strength—for mixtures with CNF, higher flexural strengths were obtained at 0.04 wt.%, with an improvement of about 25 percent over the reference mixture. The slight decrease in flexural strength in mixes with CNF contents higher than 0.04% may be due to agglomeration in the CNF particles and their poor dispersion in the cement matrix.
- tensile strength—the CNF mixtures achieved a tensile strength of 4.52 MPa, showing an improvement of about 40% over the control mixture for the 0.02% CNF admixture. The improvement in tensile strength with CNF was due to optimal dispersion through an indirect sonication process to which it was subjected before being added to the cementitious mix, leading to a denser microstructure and better crack-retention performance at the initial nano-scale level.
- morphology—the amount of non-hydrated cement is significantly lower than in CNTs and control mixtures. It may be due to the water absorption effect of CNF, which releases retarded water that helps hydrate unhydrated cement particles, and consequently strengthens the cement matrix by reducing porosity and micropores, as well as improving the strength of the cement matrix.
- X-RAY diffraction—The C-H content of the control mix was higher than that of the CNT and CNF mixes, which may explain the lower compressive strength compared to the CNT and CNF grout. C-H peaks at 2 theta were significantly higher in the CNF mix than in the CNT mix. It confirms the effect of CNF in increasing the hydration process and the calcium hydroxide content, leading to higher tensile and flexural strength and microstructure than CNT.
- atomic force microscopy—the particle size is more prominent for mixtures with CNF compared to CNT I at 1.71 μm (1.07 μm for CNT). The rib systems in the CNF blend were significantly heterogeneous compared to the CNT blend, while the C-S-H crumbs were more pronounced than in the CNT blend. It can be attributed to the effect of CNF on the hydration process, which produces a tight surface, reduces the size of pore structures, and blocks the penetration of any fluids into the cement paste. On the other hand, in CNT cement pastes, the ribbed texture essentially recognized the excellent dispersion of carbon nanotubes. It can be concluded that CNTs had a strong influence on cement composites through their physical effect, unlike CNFs, whose influence seems to be mainly due to their effect on the hydration process.

### 7.8. Haque et al.: A Comparative Investigation on the Effects of Nanocellulose from Bacteria and Plant—Based Sources for Cementitious Composites [110]

Nanocellulose: bacterial cellulose (BC) produced by *Acetobacter xylinum* bacteria and cellulose nanofibrils (CNF) from bleached sulfate hardwood pulp. Raw NC was in slurry form (1% weight solid in water).

Cement composite type: cement paste and mortar.

w/c ratio: 0.35 (cement paste), 0.5 (mortar).

Use: direct.

NC dosage: 0.05%, 0.1%, 0.3% by weight of cement.

Procedure for adding NC: cement paste—NC was added to the water and mixed at a slow speed (60 s). Cement was then added and mixed for 2 min at medium speed; mortar—NC was added to the water and mixed at a slow speed. Then, remaining at the slow speed of the mixer, cement, and sodium borosilicate were added. The speed was then changed to medium. CrI—no data available.

Components of the mixtures: cement paste—ordinary Portland cement (type I/II), water, CNFs, BC. mortar—ordinary Portland cement (type I/II), water, CNFs, BC, sodium borosilicate.

Results:

- the heat of hydration—the addition of CNF caused an increase in the heat of hydration, and the addition of BC in the early stages of hydration caused a delay in hydration. The accelerated heat flow in the case of CNF may be due to the larger surface area it creates. Despite the initial difference in the effect on hydration caused by CNF and BC additives after 7 days of curing, the authors found the same degree of hydration in both cases.
- compressive strength and flexural strength—the study showed that after 90 days of curing, the compressive and flexural strengths for both NCs resulted in strength increases of 10% and 60%, respectively. The authors determined that the most appropriate dosage should be used was 0.1%. Doses above this value cause a decrease in flexural strength for CNF and compressive strength for BC.
- thermogravimetric analysis (TGA)—compared to control samples, BC and CNF contributed to a decrease in C-H (after 7, 28, 56, and 90 days). The authors concluded that the degree of hydration after long-term curing depends on which nanocellulose was used.
- mercury intrusion porosimetry (MIP)—comparing BC and CNF, the authors found that the BC content of the material contributed to an increase in the porosity of the samples. In contrast, the addition of CNF led to an increase in total porosity but also reduced the critical pore diameter.
- dynamic vapor sorption (DVS)—when NCs were added, the amount of C-S-H in the structure increased (the structure did not change)—that is, the addition increased the specific surface area and the gel and interlayer porosity of the cement paste. The NCs caused a thickening of the matrix.
- morphology—the study showed that after the addition of NCs, BC, and CNF fibers with diameters of 60 nm and 30 nm appeared in the structure. The images showed a perfect distribution of NCs in the material’s structure.
- nanoindentation—the amount of HD CSH increased after adding NCs compared to the reference samples. The authors showed that adding 0.3%, CNF resulted in the highest amount of HD CSH.
- alkali-silica reaction (ASR)—BC did not affect ASR, while 0.1% CNF reduced ASR-induced expansion by 33%, compared to reference samples.

### 7.9. Tay et al.: Nanocellulose Reinforced Zeolite Based Geopolymer Concrete: Density Analysis through Response Surface Methodology [45]

Nanocellulose: cellulose nanofibrils (CNF) produced by ZoepNano, NC concentration was 2.0% (*w/v*) in distilled water.

Cement composite type: geopolymer foam concrete (GFC).

w/c ratio: N/A.

Use: direct.

NC dosage: 0.4 weight percent of primary geopolymer slurry.

Procedure for adding NC: NC was added together with a foaming agent to the primary geopolymer slurry. CrI—no data available.

Components of the mixtures: zeolite powder, seawater, potassium hydroxide, potassium silicate, foaming agent hydrogen peroxide, sodium lauryl ether sulfate, and benzalkonium chloride.

Results:

- water immersion—the study lasted 30 days. It showed that none of the samples made for testing were damaged or cracked. It also applies to samples with nanocellulose.
- density—the authors in the paper identified the components of the geopolymer to show, which have a significant effect on its density. Analyses showed that the addition of nanocellulose also significantly affected the density of the final material, ranging from 1.5 to 2.4 g/cm^3^. It even produced two ultra-light geopolymer foam concretes during the study, which had densities of 1.486 and 1.496 g/cm^3^.

### 7.10. Damasco et al.: Synthesis of Nanocellulose from Durian Rinds for the Preparation of a Self—Healing Smart Concrete with Augmented Mechanical Properties [111]

Nanocellulose: nanocellulose (NC) from durian rinds.

Cement composite type: self-healing smart concrete.

w/c ratio: 0.50.

Use: indirect.

NC dosage: 5.0 weight percent with respect to cement.

Procedure for adding NC: powder—SiO_2_ encapsulated with NC and UF mixed in a cementitious matrix. CrI—no data available.

Components of the mixtures: cement, coarse and fine aggregates, water, self-healing agent (SiO_2_), encapsulated with SiO_2_ urea-formaldehyde (SiUF), SiO_2_ encapsulated with NC and UF (SiUFNC).

Results:

- compressive strength and tensile strength—the strength increase was slight for SiUFNC compared to SiO_2_ (new concrete). The strengths for the healed concrete samples followed the same trend, that is, SiO_2_, SiUF, and SiUFNC increased in strength compared to the reference concrete—the highest values compared to all concretes were achieved by the concrete with SiUFNC—had a 28.6% higher strength value compared to the reference concrete.
- water absorption by capillarity—the study showed that the SiUFNC admixture most sealed the structure. After 70 days, concrete with SiUFNC had about 31% lower water absorption by capillarity than the reference concrete. The hydrophilic properties of NC likely influenced the healing process of the concrete.
- optical microscopy—in the SEM images of concrete with SiUFNC, it can be seen that the healing products filled cracks and pores in the concrete, which contributed to an increase in the mechanical parameters of the material.

### 7.11. Ramakrishnan et al.: Preparation of Nanofibrillated Cellulose and Nanocrystalline Cellulose from Surgical Cotton and Cellulose Pulp in Hot—Glycerol Medium [81]

Nanocellulose: cellulose nanocrystals (CNC), surface modified with tetraethylorthosilicate (TEOS)-TEOS-CNC.

Cement composite type: mortar.

w/c ratio: 0.4, 0.45 and 0.50.

Use: direct.

NC dosage: 0.5%, 1.0%, 1.5% by weight of cement.

Procedure for adding NC: CNC and TEOS-CNC powder dispersed in the water. CrI-CNC 97.9%.

Components of the mixtures: Portland cement, sand, water, CNC, or TEOS-CNC.

Results:

- workability—from the results presented by the authors for w/c 0.45, we can see that adding 0.5% CNC or TEOS-CNC contributed to a slight improvement in workability. However, higher doses of NC worsened the workability of the mortars.
- compressive strength—during strength testing, it was noted that the best results were achieved for a w/c of 0.45. Strength was tested after 3, 7, and 28 days. The best results were obtained for mortars with CNC and TEOS-CNC at 0.5% and 1.0%. Increasing the dosage in each case led to an increase in material strength after 28 days. Adding NCN at doses of 0.5% and 1.0% led to strength increases of 24% and 30%, respectively, and 39% and 44% for TEOS-CNC. The high aspect ratio and specific strength of CNC and the results of their even distribution in the material helped achieve higher strength values than the reference samples.
- morphology—SEM images were taken of the reference mortar and the mortar with TEOS-CNC 1.0%. The images showed that the reference mortar had more micropores in the structure. In contrast, the mortar with TEOS-CNC 1.0% was characterized by a reduced number of pores because the TEOS-CNC admixture filled the pores formed in the structure, which is also reflected in the compressive strength results.

### 7.12. Claramunt et al.: Effect of Nanocelluloses on the Microstructure and Mechanical Performance of CAC Cementitious Matrices [112]

Nanocellulose: cellulose nanocrystals (CNC) from crystalline microcellulose (CNF) from sisal pulp.

Cement composite type: cement paste.

w/c ratio: 0.30, 0.35, and 0.40.

Use: direct.

NC dosage: 0.1%, 0.2%, 0.4%, and 0.8% by weight of cement.

Procedure for adding NC: solution of NC and the superplasticizer were immersed in water and sonicated for 5 min, then cement was added. CrI—no data available.

Components of the mixtures: calcium aluminate cement, ordinary Portland cement, superplasticizer, water, CNCs, and CNFs. The samples were cured at two temperatures: 20 and 60 °C.

Results:

- modulus of rupture and modulus of elasticity—after adding either 0.4% or 0.8% nanocellulose, both values decreased compared to the control samples. It may be because the authors, with the increase in nanocellulose content, increased the w/c ratio from 0.30 to 0.35 and 0.45, respectively. It is possible that if the same w/c ratio had been maintained, as in other cases, these values would not have deteriorated. The addition of nanocellulose at a dose of 0.1% or 0.2% (CNC or CNF) improved strength parameters. In the case of NCF, only the 0.1% addition contributed to a slight improvement in the modulus of rupture. However, in the case of CNC 0.1%, the increase was significant. The addition of CNC may have contributed to enhancing the curing of calcium aluminate cement paste, which was reflected in the reduction of the degradation effect of the samples under accelerated aging.
- microstructure—during microstructure analysis, a crack-bridging effect was noted in samples with CNF. However, the low content of CNF, or its degradation, contributed to the reduction of this effect, thereby leading to a deterioration of the strength properties.
- porosity—the addition of 0.1% and 0.2% CNC increased the porosity of the samples. However, doing so seems to counteract the adverse effects of increased porosity and improve the mechanical properties of CAC pastes.

## 8. Properties of Cement Composites with Nanocellulose

Table 4 below summarizes the articles presented in Section 7.

Summarizing the information in Section 7, it can be deduced that the use of nanocellulose leads to improved properties of cement composites. However, tests with different doses and types of nanocellulose should be performed beforehand, as some combinations yield positive results and others lead to deterioration of the properties of cement composites. As well, it is important to keep in mind that not every cementitious composite will behave in the same way, for example, if nanocellulose has improved performance in mortar, it may deteriorate in concrete. Among other factors, it is influenced by the composition of the cement composite: the type of cement used, the type and composition of the aggregate including its porosity, the origin of the nanocellulose, the type of nanocellulose, the shape of the nanocellulose, the crystallinity index of the nanocellulose and so forth.

Scientists these days are looking for new methods to improve the properties of concrete or other cement composites using plant-based or recycled materials. By doing so, they want to reduce the amount of cement used in the production of cement composites and also extend the life of structures. It is very important in today’s times to strive to reduce the production of CO_2_—because cement production produces about 0.37 kg of CO_2_ for every 0.41 kg of cement [113]. Table 4 confirms that the use of an appropriate dose of nanocellulose and type can improve material properties very significantly. The use of nanocellulose surface modified with tetraethylorthosilicate (TEOS) at doses of 0.5% and 1.0% (w/c 0.45) in cement mortar results in an increase in compressive strength by39% and 44%, respectively [81]. The paper by Barnat-Hunek et al. shows that CNC in a dosage of 1.0% contributes to a reduction in the water absorption by 64.3% as well as to improve compressive strength value by 37.9% compared to the reference concrete and NCF at a dose of 1.0% results in a 34.5% increase in tensile strength. These two works and others listed in Table 4 confirm that the use of nanocellulose brings improvements in strength parameters, among other things, which is very important in the production of cement composites. The admixture of nanocellulose may contribute to the possibility of reducing the amount of cement in formulations for concrete, for example, while maintaining the same designed strength. However, at the same time, it should be remembered that the wrong choice of nanocellulose can contribute to the deterioration of performance. In some cases, increasing the nanocellulose dosage above 0.05% results in a decrease in strength increase or even a decrease below the value for reference samples [105]. Therefore, it is always necessary to carry out tests for different combinations of nanocellulose doses in order to determine the best one and to know above what dose deterioration may occur.

## 9. Further Perspectives Regarding Nanocellulose

Analyzing the latest research on nanocellulose, the authors noted that it would be appropriate to analyze further the influence of the shape, crystallinity, and dispersion of NC used in the production of cement composites. It can be noted that these three factors have a significant impact on the quality and strength parameters of the final material. Sometimes smaller doses of nanocellulose give better results than a higher content of NC added to the material, though the opposite also happens. It is essential to know the mechanism of why this happens to be able to choose the nanocellulose that will be best for use in cement composites. Strength and durability aspects are essential here, but so are economic aspects.

Adding large amounts of nanocellulose to improve the properties of the final material can be a financial barrier for some manufacturers and may not be cost-effective. The results presented in the paper [105] are consistent and indicate that the shape-uniformity of the nanocellulose has a powerful influence on the final material. Also, from the results presented, it can be concluded that the correct dispersion of NC in the material structure was achieved. In this case, the sonification of the nanocellulose in water before adding it to the cement mixture proved to be an effective way to achieve the correct dispersion. Sonification to achieve proper dispersion is also used by other authors [53,114]. It seems to be a perfect method to achieve the correct distribution of NC in the material. However, there is not always access to such equipment. It is also necessary to look at other methods, but most importantly, to identify whether the material has achieved the correct dispersion. SEM images are often insufficient because they only give a view of a small portion of the material’s structure. In addition to strength properties, the effect of nanocellulose on such parameters of cementitious composites as frost resistance [57,115] or resistance to salt crystallization should also be further identified. Frost and salts easily soluble in water negatively affect the durability of cementitious materials, so the use of nanocellulose can help protect materials from these factors.

After a literature review, the authors found no reports on using spherical or square/rectangular nanocellulose to modify cement composites. It would be necessary to conduct a detailed study and see how their use could affect the parameters of the final material. As part of the ongoing research for Szafraniec’s doctoral dissertation, square/rectangular nanocellulose was successfully produced and used to modify ordinary concrete. The results of the research indicate that this type of nanocellulose is as suitable as possible for improving the performance of concrete. However, this topic should be explored and developed further. In addition, attention should also be paid to other possibilities of using nanocellulose—not only for structural modification but also for surface modification of concrete. In the work of Barnat-Hunek et al. [116], NC was used as a material for modifying organosilicon compounds to increase the effectivity of hydrophobization.

Nanocellulose, as a recycled green material, is gaining increasing recognition in the civil engineering world. In other perspectives regarding nanocellulose, it would be necessary to fill the gaps in the literature indicated above to understand this innovative material better and apply it more effectively in cement composites.

## Figures and Tables

**Table 1:** Recently published reviews, research article, and special issues concerning cellulose as a reinforcing matrix material for polymer or fiber-cement composites.

Year	Type of Cellulose	Type of Composite Material	Type of Article	Ref.
2022	CNF	Cement Paste and Mortar	Research article	[41]
2022	CNC	Ultrahigh-Performance Fiber-Reinforced Concretes	Research article	[42]
2022	CNF	Ultra-High-Performance Concrete	Research article	[43]
2022	BNC	Self-Compacting Mortar	Research article	[44]
2022	CNF	Zeolite Based Geopolymer Concrete	Research article	[45]
2021	BNC	Concrete	Review	[46]
2021	Cellulose Fiber	Cement	Special Issue	[47]
2020	Cellulose Filaments	Ultra-High Performance Concrete	Research article	[48]
2020	Cellulose fiber	Concrete	Case study	[49]
2020	CNC, CNF, BNC, CF	Cementitious Materials	Review	[50]
2019	CNC, CNF	Cement Composites	Review	[51]
2018	Cellulose Filaments	Cement Paste and Concrete	Research article	[52]
2016	CNF	Cement	Research article	[53]
2015	Cellulose Fiber	Cement-based composites	Review	[54]
2009	Cellulose fibres	Cement Composites	Research article	[37]

**Table 4:** Summary of information contained in Section 7.

Type of NC	Source	Type of Cement Composite	w/c Ratio	Method of Use:	NC Dosage	Procedure for Adding NC	Positive Effect of NC on the Properties of the Cement Composite	Ref.
BNC	*Gluconacetobacter xylinus* bacteria	mortar	0.50	direct-powder and gel; indirect—BC coated onto the polypropylene fibers	direct—0.1%, 0.3%, and 0.5% by volume of cement; indirect—0.5%, 1.0% and, 1.5% by volume of mortar	direct—BNC added to batch water; indirect—polypropylene fibers treatment with BNCs added to mortar mix	0.3% BNC powder: the flexular strength increased by 94%; 0.3% BNC gel: increase in compressive strength by 22%; BNC powder and gel: decrease in water absorption from 6 to 37%	[83]
ONC	cotton wool	mortar	0.48	direct	0.3%, 0.6%, 1.2% and 2.4% by weight of cement	suspension of the ONCs added to premixed commercial mortar	2.4% ONC: the largest increase in compressive strength by 34% 2.4% ONC: the lowest water absorption 2.4% ONC: reduce the porosity ONC has no effect on the thermal stability	[104]
CNC	office paper waste	mortar	0.50		0.25%, 0.5%, 0.75%, 1.0% and 1.5% by weight of cement	powder—three different CNCs added as an additive to cement mixes; C1, C2, C3 for which the CrI was 79.91%, 84.23%, and 89.31%, respectively	C1 0.25%: the smallest decrease in the size of the flow diameter—5%; increased the compressive strength by 21.9% after 28 days; C1 0.75%: the flexular strength increased by 31.3%; C1 0.75% and 1.0%: the largest decrease in volume of permeable voids—14.6%; C1 1.5%: a 13.5% increase in thermal conductivity	[105]
CNC CNF	CNC-apple; CNF-carrot	concrete	0.45	direct	0.5% and 1.0% by weight of cement	water suspensions	CNC 1.0%: reduction in the water absorption by 64.3%; the largest decrease in open porosity of 48.8%; CNC 1.0%: compressive strength value increased by 37.9%; NCF 1.0%: the tensile strength increased by 34.5%; CNC 1.0%: reduction in cumulative pore volume by 40.7%; CNC 1.0%: the highest hydrophobicity for this concrete—SFE value 46.1 mJ/m^2^; CNC 1.0%: the smallest decrease in compressive strength—0.18% after 100 F-T cycles; CNC 1.0%: a more compact structure compared	[57]
CNF	bleached softwood pulp	cement paste	0.35 0.45	direct-CNF and CNF modified with nanosilica (Si-CNF)	0.025%, 0.05%, 0.1%, 0.3%, and 0.5% by weight of cement	water suspensions	CNF 0.05%: the highest increase in compressive strength after 90 days of curing—24% for w/c 0.35 and 15% for w/c 0.45; Si-CNF 0.5%: the highest increase in compressive strength after 90 days of curing—22% for w/c 0.35 and 14% for w/c 0.45; CNF 0.5% and Si-CNF 0.5%: tensile strength increased by 75% (CNF) and 55% (Si-CNF); CNF 0.025% and CNF 0.5%: increase in elastic modulus by 200% (0.025%)and 250% (0.5%)	[107]
CNC	wood pulp cotton	mortar	0.55	direct —CNCs: CNC-C-containing carboxyl groups and CNC-S-containing sulfo groups; indirect—film CNC coated onto the polypropylene fibers	direct—0.01%, 0.05%, 0.1%, 0.3% for CNC-C and CNC-S and 0.5% for CNC-S by weight of cement; indirect—0.3% volume ratio of fiber to mortar	direct-CNCs solution; indirect-polypropylene fibers coated with CNC (PPCNC)	PPCNC: compressive strength increased by 11%; CNC-C 0.05%: the compressive strength increased by 22.28%; CNC-C 0.05%: flexural strength increased by 23%; CNC affects the growth of hydrated products, changes the shape and size of hydrated crystals, and affects the compactness of the mortar structure	[108]
CNF	cotton	mortar	0.43	direct	0.02%, 0.04%, 0.06%, and 0.08% by weight of cement	solution	CNF 0.04%: increase in compressive strength by 10.65% after 28 days; CNF 0.04%: increase in flexural strengths by 25%; CNF 0.02%: improvement of about 40% in tensile strength; CNF: the amount of non-hydrated cement is significantly lower than in mixtures with CNTs and in the control mixture	[109]
BNC	*Acetobacter xylinum* bacteria	cement paste	0.35	direct	0.05%, 0.1%, 0.3% by weight of cement	slurry	CNF 0.1% and BNC 0.1%: after 90 days of curing, the compressive and flexural strengths for both NCs resulted in strength increases of 10% and 60%; CNF: an increase in total porosity, but also reduction of the critical pore diameter; CNF 0.3%: the highest amount of HD CSH; CNF 0.1%: ASR-induced expansion reduced by 33%	[110]
CNF	bleached sulfate hardwood pulp	mortar	0.50					
CNF	produced by ZoepNano	geopolymer foam concrete	N/A	direct	0.4% by weight of basic geopolymer slurry	slurry	CNF: none of the samples were damaged, or cracked after 30 days of water immersion study	[45]
NC	durian rinds	self-healing smart concrete	0.05	indirect	5.0% by weight of cement (self-healing material)	powder-SiO_2_ encapsulated with NC and UF (SiUFNC)	SiUFNC: a 28.6% higher strength value of compressive strength and tensile strength; SiUFNC: about 31% lower water absorption by capillarity; SiUFNC: the healing products filled cracks and pores in the concrete	[111]
CNC	N/A	mortar	0.40 0.45 0.50	direct	0.5%, 1.0%, 1.5% by weight of cement	CNC and TEOS-CNC (CNC surface modified with TEOS) powder dispersed in the water	CNC 0.5% and TEOS-CNC 0.5% (w/c 0.45): a slight improvement in workability; CNC 0.5% and 1.0% (w/c 0.45): compressive strength increases of 24% and 30%; TEOS-CNC 0.5% and 1.0% (w/c 0.45): compressive strength increases of 39% and 44%; TEOS-CNC 1.0%: reduced number of pores in mortar structure	[81]
CNC	crystalline microcellulose	cement paste	0.30 0.35 0.40	direct	0.1%, 0.2%, 0.4% and 0.8% by weight of cement	solution	CNC 0.1% or 0.2%: and CNF 0.1% or 0.2%: contributed to improvement in strength parameters; CNC 0.1%: significant increase of modulus of rupture; CNC: reduction of the degradation effect of the samples under accelerated aging	[112]
CNF	sisal pulp							

## Data Availability

Not applicable.
